# Supplementary material for: Phylogeny and Genetic Divergence among Sorghum Mosaic Virus Isolates Infecting Sugarcane
Source: Plants (Basel). 2023 Nov 2;12(21):3759. doi: 10.3390/plants12213759 (PMC10648118; doi:10.3390/plants12213759)
Supplement: Supplementary file 1 [file plants-12-03759-s001.zip › plants-2684281-supplementary.pdf]

Table S1. Percent identities (%) of nucleotide (low-left) and amino acid (up-right) sequences of SrMV *CP* within and between different geographical origins [a].

Geographical origins	Asia (n = 296)	South America (n = 10)
Asia	72.0~100 (74.9~100)	74.9~100
South America	71.3~100	95.5~100 (95.8~100)

[a] Amino acid sequence identities (%) of SrMV CP within geographical origins are shown in parentheses.

Table S2. Percent identities (%) of nucleotide (low-left) and amino acid (up-right) sequences of SrMV *CP* within and between host types [a].

Host types	Modern cultivar (n = 288)	Chewing cane (n = 18)
Modern cultivar	71.3~100 (74.9~100)	74.9~100
Chewing cane	71.3~100	74.8~99.8 (85.9~100)

[a] Amino acid sequence identities (%) of SrMV CP within host types are shown in parentheses.

Table S3. Information of sequences from sorghum mosaic virus (SrMV) isolates worldwide.

No.	SrMV group	Isolate	NCBI accession no.	Region/Country	Host	Reference
1	A	FJFZ012	MZ419570	FuZhou, Fujian, China	Chewing cane	He et al. (2022)
2	A	FJFZ014	MZ419571	FuZhou, Fujian, China	Chewing cane	He et al. (2022)
3	D	FJFZ024	MZ419572	FuZhou, Fujian, China	Modern cultivar	He et al. (2022)
4	A	FJFZ026	MZ419573	FuZhou, Fujian, China	Modern cultivar	He et al. (2022)
5	D	FJFZ028	MZ419574	FuZhou, Fujian, China	Modern cultivar	He et al. (2022)
6	E	FJFZ030	MZ419575	FuZhou, Fujian, China	Modern cultivar	He et al. (2022)
7	D	FJFZ035	MZ419576	FuZhou, Fujian, China	Modern cultivar	He et al. (2022)
8	C	FJFZ036	MZ419577	FuZhou, Fujian, China	Modern cultivar	He et al. (2022)
9	D	FJFZ037	MZ419578	FuZhou, Fujian, China	Modern cultivar	He et al. (2022)
10	D	FJFZ038	MZ419579	FuZhou, Fujian, China	Modern cultivar	He et al. (2022)
11	B	FJFZ041	MZ419580	FuZhou, Fujian, China	Modern cultivar	He et al. (2022)
12	C	FJFZ042	MZ419581	FuZhou, Fujian, China	Modern cultivar	He et al. (2022)
13	D	FJFZ045	MZ419582	FuZhou, Fujian, China	Modern cultivar	He et al. (2022)
14	A	FJFZ061	MZ419583	FuZhou, Fujian, China	Modern cultivar	He et al. (2022)
15	D	FJFZ064	MZ419584	FuZhou, Fujian, China	Modern cultivar	He et al. (2022)
16	E	FJSX017	MZ419585	FuZhou, Fujian, China	Modern cultivar	He et al. (2022)
17	E	FJSX023	MZ419586	FuZhou, Fujian, China	Modern cultivar	He et al. (2022)
18	E	FJSX026	MZ419587	FuZhou, Fujian, China	Modern cultivar	He et al. (2022)
19	E	FJSX027	MZ419588	FuZhou, Fujian, China	Modern cultivar	He et al. (2022)
20	D	FJZZ011	MZ419589	Zhangzhou, Fujian, China	Modern cultivar	He et al. (2022)
21	D	GDZJ203	MZ419590	Zhangzhou, Fujian, China	Modern cultivar	He et al. (2022)
22	A	GXBS060	MZ419591	Baise, Guangxi, China	Modern cultivar	He et al. (2022)
23	A	GXBS061	MZ419592	Baise, Guangxi, China	Modern cultivar	He et al. (2022)

24	A	GXBS070	MZ419593	Baise, Guangxi, China	Modern cultivar	He et al. (2022)
25	E	GXBS072	MZ419594	Baise, Guangxi, China	Modern cultivar	He et al. (2022)
26	A	GXBS074	MZ419595	Baise, Guangxi, China	Modern cultivar	He et al. (2022)
27	A	GXCZ036	MZ419596	Chongzuo, Guangxi, China	Modern cultivar	He et al. (2022)
28	E	GXFCG021	MZ419597	Fangchenggang, Guangxi, China	Modern cultivar	He et al. (2022)
29	E	GXFCG048	MZ419598	Fangchenggang, Guangxi, China	Modern cultivar	He et al. (2022)
30	A	GXLB044	MZ419599	Laibin, Guangxi, China	Modern cultivar	He et al. (2022)
31	A	GXLB045	MZ419600	Laibin, Guangxi, China	Modern cultivar	He et al. (2022)
32	A	GXLB047	MZ419601	Laibin, Guangxi, China	Modern cultivar	He et al. (2022)
33	D	GXLZ001	MZ419602	Laibin, Guangxi, China	Modern cultivar	He et al. (2022)
34	E	GXLZ002	MZ419603	Laibin, Guangxi, China	Modern cultivar	He et al. (2022)
35	E	GXLZ003	MZ419604	Laibin, Guangxi, China	Modern cultivar	He et al. (2022)
36	D	GXNN075	MZ419605	Nanning, Guangxi, China	Modern cultivar	He et al. (2022)
37	D	GZAL001	MZ419606	Anlong, Guizhou, China	Modern cultivar	He et al. (2022)
38	D	GZAL002	MZ419607	Anlong, Guizhou, China	Modern cultivar	He et al. (2022)
39	D	GZAL005	MZ419608	Anlong, Guizhou, China	Modern cultivar	He et al. (2022)
40	D	GZCH001	MZ419609	Ceheng, Guizhou, China	Modern cultivar	He et al. (2022)
41	D	GZCH002	MZ419610	Ceheng, Guizhou, China	Modern cultivar	He et al. (2022)
42	A	GZCH004	MZ419611	Ceheng, Guizhou, China	Modern cultivar	He et al. (2022)
43	D	GZCH005	MZ419612	Ceheng, Guizhou, China	Modern cultivar	He et al. (2022)
44	D	GZCH006	MZ419613	Ceheng, Guizhou, China	Modern cultivar	He et al. (2022)
45	D	GZCH007	MZ419614	Ceheng, Guizhou, China	Modern cultivar	He et al. (2022)
46	D	GZCH008	MZ419615	Ceheng, Guizhou, China	Modern cultivar	He et al. (2022)
47	D	GZCH009	MZ419616	Ceheng, Guizhou, China	Modern cultivar	He et al. (2022)
48	D	GZCH010	MZ419617	Ceheng, Guizhou, China	Modern cultivar	He et al. (2022)

49	D	GZCH011	MZ419618	Ceheng, Guizhou, China	Modern cultivar	He et al. (2022)
50	D	GZCH012	MZ419619	Ceheng, Guizhou, China	Modern cultivar	He et al. (2022)
51	D	GZCH015	MZ419620	Ceheng, Guizhou, China	Modern cultivar	He et al. (2022)
52	D	GZCH019	MZ419621	Ceheng, Guizhou, China	Modern cultivar	He et al. (2022)
53	D	GZCH022	MZ419622	Ceheng, Guizhou, China	Modern cultivar	He et al. (2022)
54	D	GZCH023	MZ419623	Ceheng, Guizhou, China	Modern cultivar	He et al. (2022)
55	D	GZCH024	MZ419624	Ceheng, Guizhou, China	Modern cultivar	He et al. (2022)
56	A	GZCH025	MZ419625	Ceheng, Guizhou, China	Modern cultivar	He et al. (2022)
57	B	GZCH031	MZ419626	Ceheng, Guizhou, China	Modern cultivar	He et al. (2022)
58	D	GZCH032	MZ419627	Ceheng, Guizhou, China	Modern cultivar	He et al. (2022)
59	A	GZCH033	MZ419628	Ceheng, Guizhou, China	Modern cultivar	He et al. (2022)
60	D	GZCH034	MZ419629	Ceheng, Guizhou, China	Modern cultivar	He et al. (2022)
61	A	GZCH035	MZ419630	Ceheng, Guizhou, China	Modern cultivar	He et al. (2022)
62	D	GZCH036	MZ419631	Ceheng, Guizhou, China	Modern cultivar	He et al. (2022)
63	D	GZCH037	MZ419632	Ceheng, Guizhou, China	Modern cultivar	He et al. (2022)
64	D	GZCH038	MZ419633	Ceheng, Guizhou, China	Modern cultivar	He et al. (2022)
65	D	GZWM002	MZ419634	Wangmo, Guizhou, China	Modern cultivar	He et al. (2022)
66	A	GZWM003	MZ419635	Wangmo, Guizhou, China	Modern cultivar	He et al. (2022)
67	A	GZWM004	MZ419636	Wangmo, Guizhou, China	Modern cultivar	He et al. (2022)
68	A	GZWM005	MZ419637	Wangmo, Guizhou, China	Modern cultivar	He et al. (2022)
69	A	GZWM006	MZ419638	Wangmo, Guizhou, China	Modern cultivar	He et al. (2022)
70	A	GZWM008	MZ419639	Wangmo, Guizhou, China	Modern cultivar	He et al. (2022)
71	A	GZWM009	MZ419640	Wangmo, Guizhou, China	Modern cultivar	He et al. (2022)
72	A	GZWM010	MZ419641	Wangmo, Guizhou, China	Modern cultivar	He et al. (2022)
73	A	GZWM011	MZ419642	Wangmo, Guizhou, China	Modern cultivar	He et al. (2022)

74	A	GZWM012	MZ419643	Wangmo, Guizhou, China	Modern cultivar	He et al. (2022)
75	A	GZWM015	MZ419644	Wangmo, Guizhou, China	Modern cultivar	He et al. (2022)
76	A	GZWM016	MZ419645	Wangmo, Guizhou, China	Modern cultivar	He et al. (2022)
77	A	GZWM021	MZ419646	Wangmo, Guizhou, China	Modern cultivar	He et al. (2022)
78	A	GZWM022	MZ419647	Wangmo, Guizhou, China	Modern cultivar	He et al. (2022)
79	D	GZWM023	MZ419648	Wangmo, Guizhou, China	Modern cultivar	He et al. (2022)
80	E	GZWM025	MZ419649	Wangmo, Guizhou, China	Modern cultivar	He et al. (2022)
81	D	GZWM027	MZ419650	Wangmo, Guizhou, China	Modern cultivar	He et al. (2022)
82	D	GZWM028	MZ419651	Wangmo, Guizhou, China	Modern cultivar	He et al. (2022)
83	D	GZWM030	MZ419652	Wangmo, Guizhou, China	Modern cultivar	He et al. (2022)
84	A	GZWM034	MZ419653	Wangmo, Guizhou, China	Modern cultivar	He et al. (2022)
85	D	GZWM035	MZ419654	Wangmo, Guizhou, China	Modern cultivar	He et al. (2022)
86	A	GZWM036	MZ419655	Wangmo, Guizhou, China	Modern cultivar	He et al. (2022)
87	A	GZWM037	MZ419656	Wangmo, Guizhou, China	Modern cultivar	He et al. (2022)
88	A	GZWM038	MZ419657	Wangmo, Guizhou, China	Modern cultivar	He et al. (2022)
89	A	GZWM039	MZ419658	Wangmo, Guizhou, China	Modern cultivar	He et al. (2022)
90	D	GZWM040	MZ419659	Wangmo, Guizhou, China	Modern cultivar	He et al. (2022)
91	A	GZWM041	MZ419660	Wangmo, Guizhou, China	Modern cultivar	He et al. (2022)
92	A	GZWM042	MZ419661	Wangmo, Guizhou, China	Modern cultivar	He et al. (2022)
93	E	GZWM043	MZ419662	Wangmo, Guizhou, China	Modern cultivar	He et al. (2022)
94	A	GZWM044	MZ419663	Wangmo, Guizhou, China	Modern cultivar	He et al. (2022)
95	E	GZWM045	MZ419664	Wangmo, Guizhou, China	Modern cultivar	He et al. (2022)
96	E	GZWM046	MZ419665	Wangmo, Guizhou, China	Modern cultivar	He et al. (2022)
97	D	GZWM047	MZ419666	Wangmo, Guizhou, China	Modern cultivar	He et al. (2022)
98	A	GZWM048	MZ419667	Wangmo, Guizhou, China	Modern cultivar	He et al. (2022)

99	A	GZWM049	MZ419668	Wangmo, Guizhou, China	Modern cultivar	He et al. (2022)
100	A	GZWM050	MZ419669	Wangmo, Guizhou, China	Modern cultivar	He et al. (2022)
101	D	GZWM051	MZ419670	Wangmo, Guizhou, China	Modern cultivar	He et al. (2022)
102	D	GZWM052	MZ419671	Wangmo, Guizhou, China	Modern cultivar	He et al. (2022)
103	A	GZWM053	MZ419672	Wangmo, Guizhou, China	Modern cultivar	He et al. (2022)
104	E	GZWM054	MZ419673	Wangmo, Guizhou, China	Modern cultivar	He et al. (2022)
105	A	GZWM055	MZ419674	Wangmo, Guizhou, China	Modern cultivar	He et al. (2022)
106	A	GZWM056	MZ419675	Wangmo, Guizhou, China	Modern cultivar	He et al. (2022)
107	E	GZWM057	MZ419676	Wangmo, Guizhou, China	Modern cultivar	He et al. (2022)
108	A	GZWM058	MZ419677	Wangmo, Guizhou, China	Modern cultivar	He et al. (2022)
109	A	GZWM059	MZ419678	Wangmo, Guizhou, China	Modern cultivar	He et al. (2022)
110	A	GZWM060	MZ419679	Wangmo, Guizhou, China	Modern cultivar	He et al. (2022)
111	A	GZWM062	MZ419680	Wangmo, Guizhou, China	Modern cultivar	He et al. (2022)
112	A	GZWM063	MZ419681	Wangmo, Guizhou, China	Modern cultivar	He et al. (2022)
113	A	GZXY002	MZ419682	Xingyi, Guizhou, China	Modern cultivar	He et al. (2022)
114	D	GZXY003	MZ419683	Xingyi, Guizhou, China	Modern cultivar	He et al. (2022)
115	D	GZXY005	MZ419684	Xingyi, Guizhou, China	Modern cultivar	He et al. (2022)
116	D	GZXY006	MZ419685	Xingyi, Guizhou, China	Modern cultivar	He et al. (2022)
117	A	GZXY007	MZ419686	Xingyi, Guizhou, China	Modern cultivar	He et al. (2022)
118	A	GZXY008	MZ419687	Xingyi, Guizhou, China	Modern cultivar	He et al. (2022)
119	A	GZXY011	MZ419688	Xingyi, Guizhou, China	Modern cultivar	He et al. (2022)
120	A	GZXY012	MZ419689	Xingyi, Guizhou, China	Modern cultivar	He et al. (2022)
121	A	GZXY018	MZ419690	Xingyi, Guizhou, China	Modern cultivar	He et al. (2022)
122	B	GZXY023	MZ419691	Xingyi, Guizhou, China	Modern cultivar	He et al. (2022)
123	A	GZXY026	MZ419692	Xingyi, Guizhou, China	Modern cultivar	He et al. (2022)

124	A	GZXY028	MZ419693	Xingyi, Guizhou, China	Modern cultivar	He et al. (2022)
125	A	GZXY030	MZ419694	Xingyi, Guizhou, China	Modern cultivar	He et al. (2022)
126	A	GZXY031	MZ419695	Xingyi, Guizhou, China	Modern cultivar	He et al. (2022)
127	A	GZXY032	MZ419696	Xingyi, Guizhou, China	Modern cultivar	He et al. (2022)
128	A	GZXY033	MZ419697	Xingyi, Guizhou, China	Modern cultivar	He et al. (2022)
129	A	GZXY034	MZ419698	Xingyi, Guizhou, China	Modern cultivar	He et al. (2022)
130	D	GZXY035	MZ419699	Xingyi, Guizhou, China	Modern cultivar	He et al. (2022)
131	D	GZXY036	MZ419700	Xingyi, Guizhou, China	Modern cultivar	He et al. (2022)
132	D	GZXY037	MZ419701	Xingyi, Guizhou, China	Modern cultivar	He et al. (2022)
133	D	GZXY041	MZ419702	Xingyi, Guizhou, China	Modern cultivar	He et al. (2022)
134	D	GZXY042	MZ419703	Xingyi, Guizhou, China	Modern cultivar	He et al. (2022)
135	D	GZXY044	MZ419704	Xingyi, Guizhou, China	Modern cultivar	He et al. (2022)
136	D	GZXY045	MZ419705	Xingyi, Guizhou, China	Modern cultivar	He et al. (2022)
137	D	GZXY046	MZ419706	Xingyi, Guizhou, China	Modern cultivar	He et al. (2022)
138	D	GZXY047	MZ419707	Xingyi, Guizhou, China	Modern cultivar	He et al. (2022)
139	A	GZXY051	MZ419708	Xingyi, Guizhou, China	Modern cultivar	He et al. (2022)
140	D	GZXY052	MZ419709	Xingyi, Guizhou, China	Modern cultivar	He et al. (2022)
141	A	GZXY053	MZ419710	Xingyi, Guizhou, China	Modern cultivar	He et al. (2022)
142	D	GZXY061	MZ419711	Xingyi, Guizhou, China	Modern cultivar	He et al. (2022)
143	D	GZXY062	MZ419712	Xingyi, Guizhou, China	Modern cultivar	He et al. (2022)
144	A	HNSY002	MZ419713	Sanya, Hainan, China	Modern cultivar	He et al. (2022)
145	A	HNSY003	MZ419714	Sanya, Hainan, China	Modern cultivar	He et al. (2022)
146	D	HNSY005	MZ419715	Sanya, Hainan, China	Modern cultivar	He et al. (2022)
147	D	HNSY007	MZ419716	Sanya, Hainan, China	Modern cultivar	He et al. (2022)
148	D	HNSY009	MZ419717	Sanya, Hainan, China	Modern cultivar	He et al. (2022)

149	A	HNSY010	MZ419718	Sanya, Hainan, China	Modern cultivar	He et al. (2022)
150	E	HNSY014	MZ419719	Sanya, Hainan, China	Modern cultivar	He et al. (2022)
151	A	HNSY015	MZ419720	Sanya, Hainan, China	Modern cultivar	He et al. (2022)
152	B	HNWZS001	MZ419721	Wuzhishan, Hainan, China	Modern cultivar	He et al. (2022)
153	B	HNWZS003	MZ419722	Wuzhishan, Hainan, China	Modern cultivar	He et al. (2022)
154	B	HNWZS004	MZ419723	Wuzhishan, Hainan, China	Modern cultivar	He et al. (2022)
155	C	HNWZS007	MZ419724	Wuzhishan, Hainan, China	Modern cultivar	He et al. (2022)
156	A	HNWZS009	MZ419725	Wuzhishan, Hainan, China	Modern cultivar	He et al. (2022)
157	A	HNWZS010	MZ419726	Wuzhishan, Hainan, China	Modern cultivar	He et al. (2022)
158	D	HNWZS014	MZ419727	Wuzhishan, Hainan, China	Modern cultivar	He et al. (2022)
159	B	HNWZS016	MZ419728	Wuzhishan, Hainan, China	Modern cultivar	He et al. (2022)
160	D	HNWZS017	MZ419729	Wuzhishan, Hainan, China	Modern cultivar	He et al. (2022)
161	A	HNWZS025	MZ419730	Wuzhishan, Hainan, China	Modern cultivar	He et al. (2022)
162	B	HNWZS027	MZ419731	Wuzhishan, Hainan, China	Modern cultivar	He et al. (2022)
163	A	HNWZS029	MZ419732	Wuzhishan, Hainan, China	Modern cultivar	He et al. (2022)
164	A	HNWZS031	MZ419733	Wuzhishan, Hainan, China	Modern cultivar	He et al. (2022)
165	A	HNWZS032	MZ419734	Wuzhishan, Hainan, China	Modern cultivar	He et al. (2022)
166	A	HNWZS033	MZ419735	Wuzhishan, Hainan, China	Modern cultivar	He et al. (2022)
167	D	HNWZS034	MZ419736	Wuzhishan, Hainan, China	Modern cultivar	He et al. (2022)
168	A	HNWZS038	MZ419737	Wuzhishan, Hainan, China	Modern cultivar	He et al. (2022)
169	B	HNWZS039	MZ419738	Wuzhishan, Hainan, China	Modern cultivar	He et al. (2022)
170	B	HNWZS040	MZ419739	Wuzhishan, Hainan, China	Modern cultivar	He et al. (2022)
171	B	HNWZS041	MZ419740	Wuzhishan, Hainan, China	Modern cultivar	He et al. (2022)
172	A	SCJK001	MZ419741	Neijiang, Sichuan, China	Modern cultivar	He et al. (2022)
173	A	SCJK002	MZ419742	Neijiang, Sichuan, China	Modern cultivar	He et al. (2022)

174	D	SCJK003	MZ419743	Neijiang, Sichuan, China	Modern cultivar	He et al. (2022)
175	D	SCJK004	MZ419744	Neijiang, Sichuan, China	Modern cultivar	He et al. (2022)
176	D	SCJK005	MZ419745	Neijiang, Sichuan, China	Modern cultivar	He et al. (2022)
177	D	SCJK006	MZ419746	Neijiang, Sichuan, China	Modern cultivar	He et al. (2022)
178	D	SCJK007	MZ419747	Neijiang, Sichuan, China	Modern cultivar	He et al. (2022)
179	A	SCJK011	MZ419748	Neijiang, Sichuan, China	Modern cultivar	He et al. (2022)
180	D	SCJK034	MZ419749	Neijiang, Sichuan, China	Modern cultivar	He et al. (2022)
181	D	SCJK035	MZ419750	Neijiang, Sichuan, China	Modern cultivar	He et al. (2022)
182	C	SCJK037	MZ419751	Neijiang, Sichuan, China	Modern cultivar	He et al. (2022)
183	D	SCJK038	MZ419752	Neijiang, Sichuan, China	Modern cultivar	He et al. (2022)
184	D	SCJK039	MZ419753	Neijiang, Sichuan, China	Modern cultivar	He et al. (2022)
185	D	SCJK040	MZ419754	Neijiang, Sichuan, China	Modern cultivar	He et al. (2022)
186	C	SCJK043	MZ419755	Neijiang, Sichuan, China	Modern cultivar	He et al. (2022)
187	C	SCMX074	MZ419756	Neijiang, Sichuan, China	Modern cultivar	He et al. (2022)
188	D	SCMX079	MZ419757	Neijiang, Sichuan, China	Modern cultivar	He et al. (2022)
189	D	SCMX083	MZ419758	Neijiang, Sichuan, China	Modern cultivar	He et al. (2022)
190	D	SCMX085	MZ419759	Neijiang, Sichuan, China	Modern cultivar	He et al. (2022)
191	D	SCMX096	MZ419760	Neijiang, Sichuan, China	Modern cultivar	He et al. (2022)
192	D	SCMX097	MZ419761	Neijiang, Sichuan, China	Modern cultivar	He et al. (2022)
193	B	SCSZ001	MZ419762	Neijiang, Sichuan, China	Modern cultivar	He et al. (2022)
194	A	SCSZ002	MZ419763	Neijiang, Sichuan, China	Modern cultivar	He et al. (2022)
195	D	SCSZ003	MZ419764	Neijiang, Sichuan, China	Modern cultivar	He et al. (2022)
196	A	SCSZ005	MZ419765	Neijiang, Sichuan, China	Modern cultivar	He et al. (2022)
197	A	SCSZ006	MZ419766	Neijiang, Sichuan, China	Modern cultivar	He et al. (2022)
198	A	SCSZ008	MZ419767	Neijiang, Sichuan, China	Modern cultivar	He et al. (2022)

199	A	SCSZ009	MZ419768	Neijiang, Sichuan, China	Modern cultivar	He et al. (2022)
200	D	SCSZ010	MZ419769	Neijiang, Sichuan, China	Modern cultivar	He et al. (2022)
201	D	SCSZ011	MZ419770	Neijiang, Sichuan, China	Modern cultivar	He et al. (2022)
202	B	SCSZ012	MZ419771	Neijiang, Sichuan, China	Modern cultivar	He et al. (2022)
203	D	SCSZ013	MZ419772	Neijiang, Sichuan, China	Modern cultivar	He et al. (2022)
204	B	SCSZ014	MZ419773	Neijiang, Sichuan, China	Modern cultivar	He et al. (2022)
205	B	SCSZ015	MZ419774	Neijiang, Sichuan, China	Modern cultivar	He et al. (2022)
206	B	SCSZ016	MZ419775	Neijiang, Sichuan, China	Modern cultivar	He et al. (2022)
207	A	SCSZ017	MZ419776	Neijiang, Sichuan, China	Modern cultivar	He et al. (2022)
208	A	SCSZ019	MZ419777	Neijiang, Sichuan, China	Modern cultivar	He et al. (2022)
209	A	SCSZ020	MZ419778	Neijiang, Sichuan, China	Modern cultivar	He et al. (2022)
210	A	SCSZ021	MZ419779	Neijiang, Sichuan, China	Modern cultivar	He et al. (2022)
211	A	SCSZ022	MZ419780	Neijiang, Sichuan, China	Modern cultivar	He et al. (2022)
212	B	SCWY007	MZ419781	Neijiang, Sichuan, China	Modern cultivar	He et al. (2022)
213	C	SCWY008	MZ419782	Neijiang, Sichuan, China	Modern cultivar	He et al. (2022)
214	B	SCWY009	MZ419783	Neijiang, Sichuan, China	Modern cultivar	He et al. (2022)
215	B	SCWY010	MZ419784	Neijiang, Sichuan, China	Modern cultivar	He et al. (2022)
216	C	SCWY011	MZ419785	Neijiang, Sichuan, China	Modern cultivar	He et al. (2022)
217	B	SCWY012	MZ419786	Neijiang, Sichuan, China	Modern cultivar	He et al. (2022)
218	B	SCWY014	MZ419787	Neijiang, Sichuan, China	Modern cultivar	He et al. (2022)
219	C	SCZZ002	MZ419788	Neijiang, Sichuan, China	Modern cultivar	He et al. (2022)
220	D	SCZZ003	MZ419789	Neijiang, Sichuan, China	Modern cultivar	He et al. (2022)
221	D	SCZZ004	MZ419790	Neijiang, Sichuan, China	Modern cultivar	He et al. (2022)
222	C	SCZZ005	MZ419791	Neijiang, Sichuan, China	Modern cultivar	He et al. (2022)
223	D	SCZZ006	MZ419792	Neijiang, Sichuan, China	Modern cultivar	He et al. (2022)

224	C	SCZZ007	MZ419793	Neijiang, Sichuan, China	Modern cultivar	He et al. (2022)
225	D	SCZZ008	MZ419794	Neijiang, Sichuan, China	Modern cultivar	He et al. (2022)
226	C	SCZZ010	MZ419795	Neijiang, Sichuan, China	Modern cultivar	He et al. (2022)
227	D	SCZZ011	MZ419796	Neijiang, Sichuan, China	Modern cultivar	He et al. (2022)
228	D	SCZZ012	MZ419797	Neijiang, Sichuan, China	Modern cultivar	He et al. (2022)
229	C	SCZZ013	MZ419798	Neijiang, Sichuan, China	Modern cultivar	He et al. (2022)
230	D	SCZZ014	MZ419799	Neijiang, Sichuan, China	Modern cultivar	He et al. (2022)
231	C	SCZZ015	MZ419800	Neijiang, Sichuan, China	Modern cultivar	He et al. (2022)
232	C	SCZZ016	MZ419801	Neijiang, Sichuan, China	Modern cultivar	He et al. (2022)
233	C	SCZZ017	MZ419802	Neijiang, Sichuan, China	Modern cultivar	He et al. (2022)
234	A	SCZZ018	MZ419803	Neijiang, Sichuan, China	Modern cultivar	He et al. (2022)
235	A	SCZZ019	MZ419804	Neijiang, Sichuan, China	Modern cultivar	He et al. (2022)
236	A	SCZZ020	MZ419805	Neijiang, Sichuan, China	Modern cultivar	He et al. (2022)
237	A	SCZZ021	MZ419806	Neijiang, Sichuan, China	Modern cultivar	He et al. (2022)
238	A	SCZZ022	MZ419807	Neijiang, Sichuan, China	Modern cultivar	He et al. (2022)
239	A	SCZZ023	MZ419808	Neijiang, Sichuan, China	Modern cultivar	He et al. (2022)
240	A	SCZZ024	MZ419809	Neijiang, Sichuan, China	Modern cultivar	He et al. (2022)
241	D	SCZZ026	MZ419810	Neijiang, Sichuan, China	Modern cultivar	He et al. (2022)
242	D	SCZZ027	MZ419811	Neijiang, Sichuan, China	Modern cultivar	He et al. (2022)
243	D	SCZZ028	MZ419812	Neijiang, Sichuan, China	Modern cultivar	He et al. (2022)
244	C	SCZZ029	MZ419813	Neijiang, Sichuan, China	Modern cultivar	He et al. (2022)
245	D	SCZZ030	MZ419814	Neijiang, Sichuan, China	Modern cultivar	He et al. (2022)
246	D	SCZZ031	MZ419815	Neijiang, Sichuan, China	Modern cultivar	He et al. (2022)
247	D	SCZZ032	MZ419816	Neijiang, Sichuan, China	Modern cultivar	He et al. (2022)
248	C	SCZZ033	MZ419817	Neijiang, Sichuan, China	Modern cultivar	He et al. (2022)

249	D	SCZZ034	MZ419818	Neijiang, Sichuan, China	Modern cultivar	He et al. (2022)
250	D	SCZZ036	MZ419819	Neijiang, Sichuan, China	Modern cultivar	He et al. (2022)
251	B	YNBS020	MZ419820	Baoshan, Yunnan, China	Modern cultivar	He et al. (2022)
252	D	YNBS021	MZ419821	Baoshan, Yunnan, China	Modern cultivar	He et al. (2022)
253	B	YNDH031	MZ419822	Dehongzhou, Yunnan, China	Modern cultivar	He et al. (2022)
254	A	ZJWL001	MZ419823	Wenling, Zhejiang, China	Modern cultivar	He et al. (2022)
255	A	ZJWL002	MZ419824	Wenling, Zhejiang, China	Modern cultivar	He et al. (2022)
256	A	ZJWL005	MZ419825	Wenling, Zhejiang, China	Modern cultivar	He et al. (2022)
257	A	ZJWL035	MZ419826	Wenling, Zhejiang, China	Modern cultivar	He et al. (2022)
258	E	ZJWZ019	MZ419827	Wenzhou, Zhejiang, China	Chewing cane	He et al. (2022)
259	A	ZJWZ020	MZ419828	Wenzhou, Zhejiang, China	Chewing cane	He et al. (2022)
260	A	ZJWZ025	MZ419829	Wenzhou, Zhejiang, China	Chewing cane	He et al. (2022)
261	A	ZJWZ026	MZ419830	Wenzhou, Zhejiang, China	Chewing cane	He et al. (2022)
262	A	ZJYW001	MZ419831	Yiwu, Zhejiang, China	Modern cultivar	He et al. (2022)
263	A	ZJYW002	MZ419832	Yiwu, Zhejiang, China	Modern cultivar	He et al. (2022)
264	A	ZJYW006	MZ419833	Yiwu, Zhejiang, China	Modern cultivar	He et al. (2022)
265	D	ZJYW007	MZ419834	Yiwu, Zhejiang, China	Modern cultivar	He et al. (2022)
266	F	YN13	FM997900	Yunnan, China, China	Chewing cane	Wang et al. (2010)
267	F	YN12	FM997899	Yunnan, China, China	Chewing cane	Wang et al. (2010)
268	D	YN11	FM997898	Yunnan, China, China	Chewing cane	Wang et al. (2010)
269	B	YN16	FM997903	Yunnan, China, China	Modern cultivar	Wang et al. (2010)
270	A	YN18	FM997905	Yunnan, China, China	Modern cultivar	Wang et al. (2010)
271	B	YN14	FM997901	Yunnan, China, China	Modern cultivar	Wang et al. (2010)
272	F	YN21	FM997908	Yunnan, China, China	Modern cultivar	Wang et al. (2010)
273	F	YN22	FM997909	Yunnan, China, China	Modern cultivar	Wang et al. (2010)

274	D	YN-lc-1	KC179678	Yunnan, China, China	Modern cultivar	Zhang et al. (2015)
275	D	YN-lc-2	KC179679	Yunnan, China, China	Modern cultivar	Zhang et al. (2015)
276	A	GD-gz-sh1	DQ991392	Guangdong, China	Modern cultivar	Xu et al. (2008)
277	A	GD-gz-sh2	DQ991393	Guangdong, China	Modern cultivar	Xu et al. (2008)
278	E	GD-gz-sh3	DQ922903	Guangdong, China	Modern cultivar	Xu et al. (2008)
279	E	GD-wy-sh1	DQ227695	Guangdong, China	Modern cultivar	Xu et al. (2008)
280	A	GD-wy-sh2	EF583924	Guangdong, China	Modern cultivar	Xu et al. (2008)
281	A	GD-bl-so	DQ991390	Guangdong, China	Chewing cane	Xu et al. (2008)
282	A	GD-gz-so	DQ991391	Guangdong, China	Chewing cane	Xu et al. (2008)
283	E	GX-nn-sh1	EF419178	Guangxi, China, China	Modern cultivar	Xu et al. (2008)
284	D	GX-nn-sh2	EF419179	Guangxi, China, China	Modern cultivar	Xu et al. (2008)
285	C	GX-nn-sh3	EF419180	Guangxi, China, China	Modern cultivar	Xu et al. (2008)
286	A	GX	KJ541740	Guangxi, China, China	Modern cultivar	Zhou et al. (2014)
287	B	GX-gl-10	KC179677	Guangxi, China, China	Chewing cane	Zhang et al. (2015)
288	D	HN-lg-1	KC179638	Hainan, China, China	Modern cultivar	Zhang et al. (2015)
289	A	LH	AJ310196	Zhejiang, China, China	Modern cultivar	Chen et al. (2002)
290	A	LP	AJ310195	Zhejiang, China, China	Modern cultivar	Chen et al. (2002)
291	A	XgS	AJ310194	Zhejiang, China, China	Modern cultivar	Chen et al. (2002)
292	A	XoS	AJ310197	Zhejiang, China, China	Modern cultivar	Chen et al. (2002)
293	A	YH	AJ310198	Zhejiang, China, China	Modern cultivar	Chen et al. (2002)
294	D	hz	AJ421470	Zhejiang, China, China	Modern cultivar	Chen et al. (2002)
295	A	VN/SC5	DQ925433	Vietnam	Chewing cane	Ha et al. (2008)
296	A	VN/SC6	DQ925434	Vietnam	Chewing cane	Ha et al. (2008)
297	D	SCI	U57359	USA	Modern cultivar	Zhou et al. (2007)
298	D	SCM	U57360	USA	Modern cultivar	Zhou et al. (2007)

299	D	SCH	U07219	USA	Modern cultivar	Yang et al. (1997)
300	D	Louis	EF078962	USA	Modern cultivar	Grisham et al. (2007)
301	D	TUC-h1	EU189035	Argentina	Modern cultivar	GenBank
302	D	TUC-m2	EU189040	Argentina	Modern cultivar	GenBank
303	D	TUC-h3	EU189037	Argentina	Modern cultivar	Perera et al. (2009)
304	D	TUC-m1	EU189039	Argentina	Modern cultivar	Perera et al. (2009)
305	D	TUC-i2	EU189042	Argentina	Modern cultivar	Perera et al. (2009)
306	D	H	U57358	USA	Modern cultivar	Zhou et al. (2007)
307	Outgroup	SCMV-HZ	NC_003398	Zhejiang, China	Maize	GenBank

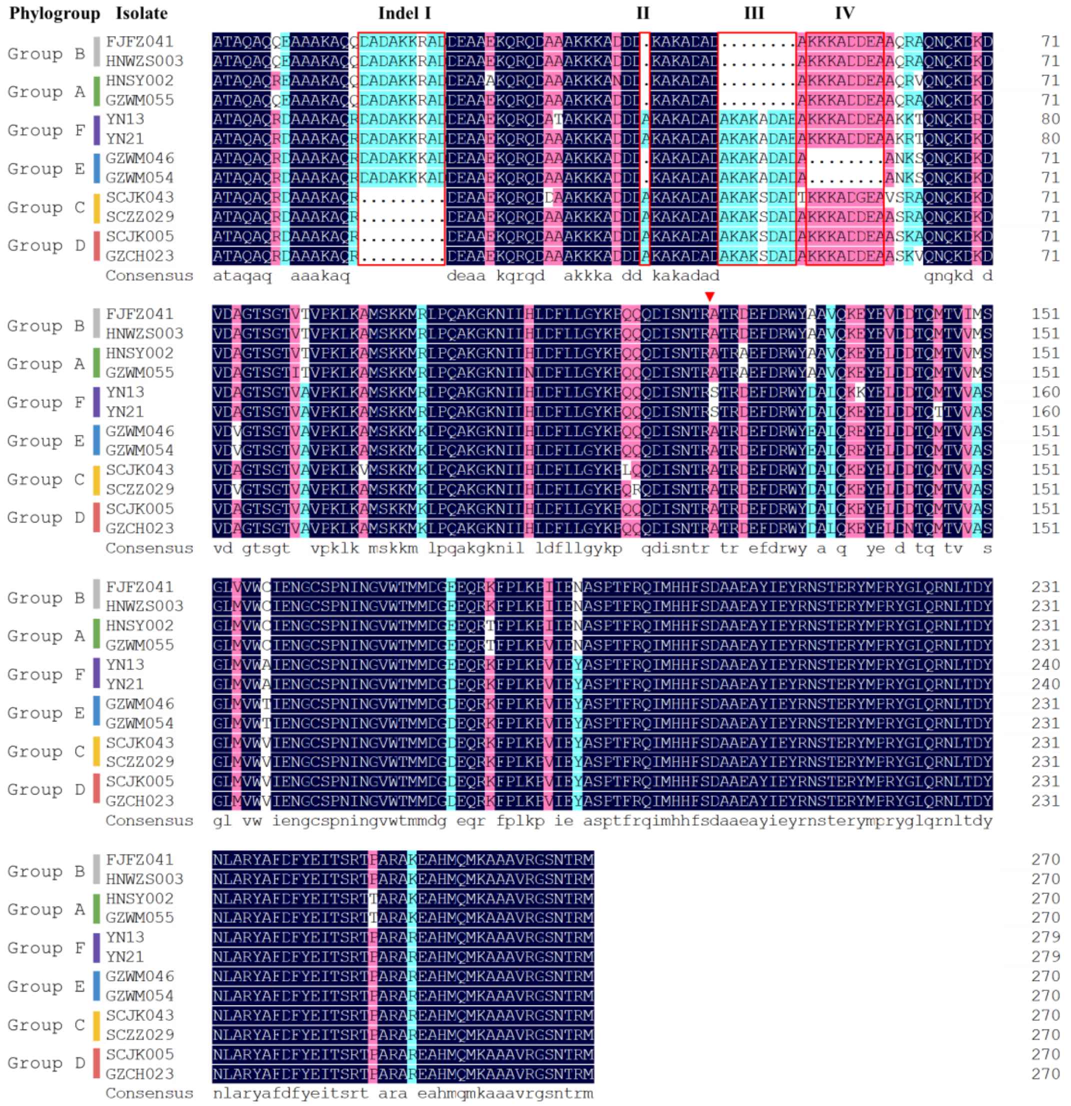

Figure S1. Insertion/deletion (InDel) and site mutation among amino acid sequences of SrMV CP. Twelve representative CP sequences (two sequences in each phylogroup) were selected for the alignment by DNAMAN version 6 software. Insertion/deletion (InDel) is showed in red boxes. A unique site mutation of SrMV CP between SrMV-F and other groups is marked with a solid triangle.
